# MyUMC Patient Portal: patient and professional perspectives

**Published:** 2012-06-15

**Authors:** Wynand J.G Ros

**Affiliations:** Julius Centre, UMC Utrecht, The Netherlands

**Keywords:** e-health, patient portal, patient experiences, substitution, usability

## Abstract

**Introduction:**

In 2010 the UMC Utrecht started with the introduction of patient portals for patients with chronic conditions. The evaluation concerned portals of three patient groups: adults with HIV, adolescents with CF and parents of children with CF. The portals had six modules: ‘overview treatment appointments’, ‘overview of medication and test results’, ‘e-consult (a-synchronous)’, ‘request for medical prescription’, ‘diaries’ and ‘questionnaires’.

**Aim and objectives:**

To evaluate patient’s use, their experiences and to detect factors that influence use and appreciation of the portal.

**Method:**

One hundred and nine patients were followed during seven months. They completed a digital questionnaire at baseline, and at 2 and 7 months. The questionnaire contains items with fixed response categories on personal characteristics (including internet use and computer skills), actual use of the portal (frequency and modules), total websites’ ease-of-use (attractiveness, controllability, efficiency, helpfulness and learnability with WAMMI), patients’ experiences with the modules and overall appreciation of the portal (10-point scale). Two open questions were add on specific positive and specific negative experiences.

**Results:**

Most respondents were regular (almost daily) users of the internet and considered themselves competent computer users. Over 80% of the patients used the portal at least once. More than half of them used the portal recently, i.e. the last month. Reasons for not using the portal were lack of actual health problems, problems with login procedures, too busy with other things. Related to regular care, most patients used the portal complementary to regular care for the same questions. However, about half of the patients stated that they used the portal as a substitution for regular consultations (face to face or telephone). On the other hand, one-third stated that they used the portal in addition to regular care. The users find the portal useful and easy to use. Almost all users visited the modules ‘medication overview and test results’ (92%) and ‘overview treatment appointments’ (82%). Also the modules ‘e-consult’ (58%) and ‘request for medical prescription’ (35%) were well attended. There were differences between patient groups. Adults with HIV evaluate the module ‘medication overview and test results’ as most important; parents of children with CF the module ‘e-consult’. Patient’s characteristics were not related to the use of the portal. Websites’ ease-to-use and overall appreciation of the portal were related to actual use. The results of the open questions show that patients appreciate the permanent (24/7) accessibility of the portal. They do not have to call several times to the hospital. Moreover, they do not have to feel troubled anymore to disturb their health care professionals for non urgent matters.

**Conclusions:**

Patients appreciate the availability of a patient portal, especially for non-urgent matters. They use a portal instead of regular contacts, but also as a source of additional information. Websites’ ease-to-use and overall appreciation are related to actual use. Age and gender are not related to actual use, however, disease-related characteristics are.

